# 

**Published:** 1886-02

**Authors:** 


### CONTENTS:

ART. I—Report on Dental Literature and Dental Education. By W. W. H. Thackston, M D., D. D. S .......... 433
ART. II—The Odonto-Chirurgical Society of Scotland .......... 441
ART. III—The Arsenical Treatment of Tooth-Pulps By A. Morsman, M. D., D. D. S 453
ART. IV—Right and Left-Handedness .......... 464
ART. V—Exsection of Inferior Dental Nerve, for Relief of Paroxysms in Lower Jaw. By L. P. Dottere, D. D. S .......... 467
Ninth International Medical Congress .......... 468
University of California .......... 469

### EDITORIAL, ETC.

The Annual Commencement of the University of Maryland, Dental Department ... 470
Dr. Oliver Wendell Holmes' Toast at the Dinner of the New York Odontological Society .......... 471
Opposing Baltimore Medical Colleges—Correction .......... 472

### BIBLIOGRAPHICAL.

Recommendations of Professor Gorgas Works .......... 473

Concerning Dr. Gorgas' "Series of Questions for Dental Students." .......... 476

Tabulae Anatomical Osteologiae .......... 478

Vick's Floral Guide for 1886 .......... 479

### MONTHLY SUMMARY.

Extraordinary Effects of Drugs .......... 479

